# Supplementary material for: Comparative analysis of technological fitness and coherence at different geographical scales
Source: PLoS One. 2025 Aug 13;20(8):e0329746. doi: 10.1371/journal.pone.0329746 (PMC12349728; doi:10.1371/journal.pone.0329746)
Supplement: S1 File — (PDF) [file pone.0329746.s001.pdf]

Supplementary Information

Comparative Analysis of Technology Fitness and Coherence at different geographical scales

Matteo Straccamore, Matteo Bruno, and Andrea Tacchella

Contents

1 Patents information **3**

2 Binarization process **3**

3 OLS regression comparison **6**

1 Patents information

Using the database provided by De Rassenfosse et al. [1], we can determine the geographical locations of approximately 18.9 million patent applicants. The objective of this study is to create a dataset comprising early patent applications from various parts of the world and organize it based on the applicants' locations. This dataset enables us to identify the origin of patented inventions and locate centers of innovation. Such information proves valuable for analyzing the geography of innovation and understanding the spatial distribution of patented inventions. Policymakers interested in firms' location decisions and the migration patterns of highly skilled workers can also benefit from this data. The authors accomplished geolocation by associating postal codes of applicant addresses with latitude and longitude coordinates, thereby determining countries, regions, and cities/metropolitan areas. They obtained postal code information by matching addresses found in patent applications within the PATSTAT database and other sources, which provide the corresponding postal codes based on these address codes.
Here are some notable features of this database:

- The patents included in this dataset represent the initial applications for respective inventions.
- Some patents have multiple geolocations due to collaborative efforts involving multiple applicants. However, it should be noted that after assigning patents to their respective metropolitan areas, only around 2% of the total patents have multiple geolocations.
- Each patent is associated with one or more CPC 4-digit technology codes. In cases where patents have multiple technology codes, we retain all of them. This approach adds minimal noise to the data since the categories are already highly specific.
- As this database focuses on the first application, the applicant's position remains consistent even if multiple patent offices are involved.
- The geolocalization of all patents in the De Rassenfosse database utilizes information from various sources, including PATSTAT, WIPO, REGPAT, and patent offices in Japan, China, Germany, France, and the United Kingdom.

2 Binarization process

As described in the main text, the first objective is to generate the bipartite $\mathbf{M}^y$ that links the three different geographical areas to their respective technologies. In this work, we decided not to work by calculating the Revealed Comparative Advantage (RCA) [2]. Although the calculation of the RCA has proven to be excellent for studies concerning the export of products [2, 3, 4, 5], in our work its application led us to questionable results, such as the United States in the bottom of the Fitness ranking, or the Tokyo metropolitan area in the middle of the respective ranking. The problems concerning the RCA have already been discussed in [6]. In our case, the problem arises when it is calculated by comparing counts, which range from very low values, such as 1, to values of very high orders of magnitude, such as 10000; problem not present in the case of exports at country level.

To avoid this, in this paper we construct the $\mathbf{M}^y$-matrices using an innovative methodology involving statistical tests to validate the number of technologies present in an entity's patents. We start with two preliminary matrices: $\mathbf{V}^y$, linking entities e to their patents p, and $\mathbf{B}^y$, linking patents to their corresponding technologies t. In these matrices, $V_{p,e} = 1$ (or 0) indicates whether patent p belongs to entity e, and $B_{p,t} = 1$ (or 0) if patent p includes technology code t.First, we count the number of observations, i.e. the number of times the technology t falls into the entity e. In Fig. 1 we show a pictorial example of the observation matrix $\mathbf{OBS}^y$ construction. We simply compute

$$OBS^y_{e,t} = \sum_p V^y_{p,e} B^y_{p,t}$$

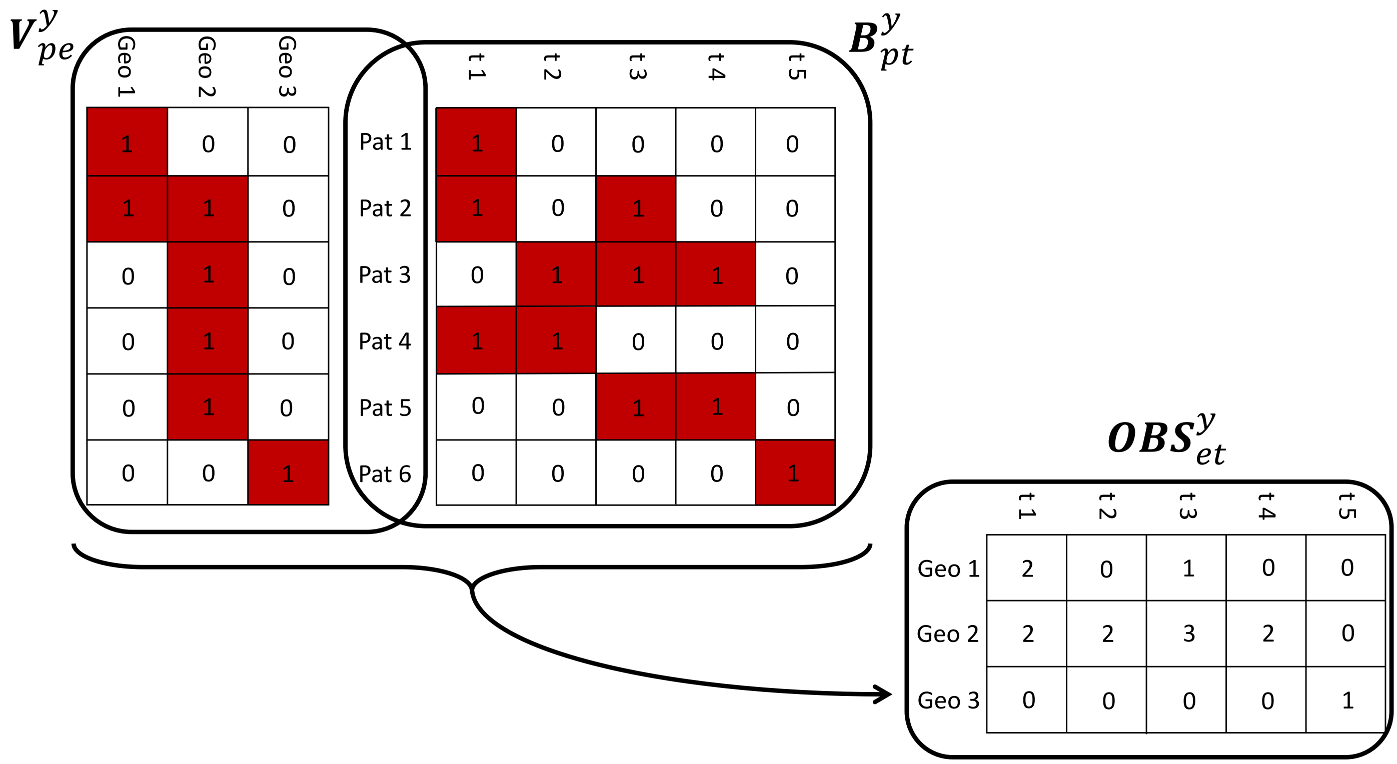

Figure 1: **Observation matrix (OBS) construction process.** In This Figure, we show how we compute the $\mathbf{OBS}^y$ matrix for each 5-time window y. We start from the bipartites $\mathbf{V}^y$ and $\mathbf{B}^y$, which elements $V^y_{p,e}$ and $B^y_{p,t}$ are 1 if an entity e, or patent p, is connected to a patent p, or technology code t, respectively. Finally, $\mathbf{OBS}^y$ is computed counting the common elements: $OBS^y_{e,t} = \sum_p V^y_{p,e} B^y_{p,t}$.

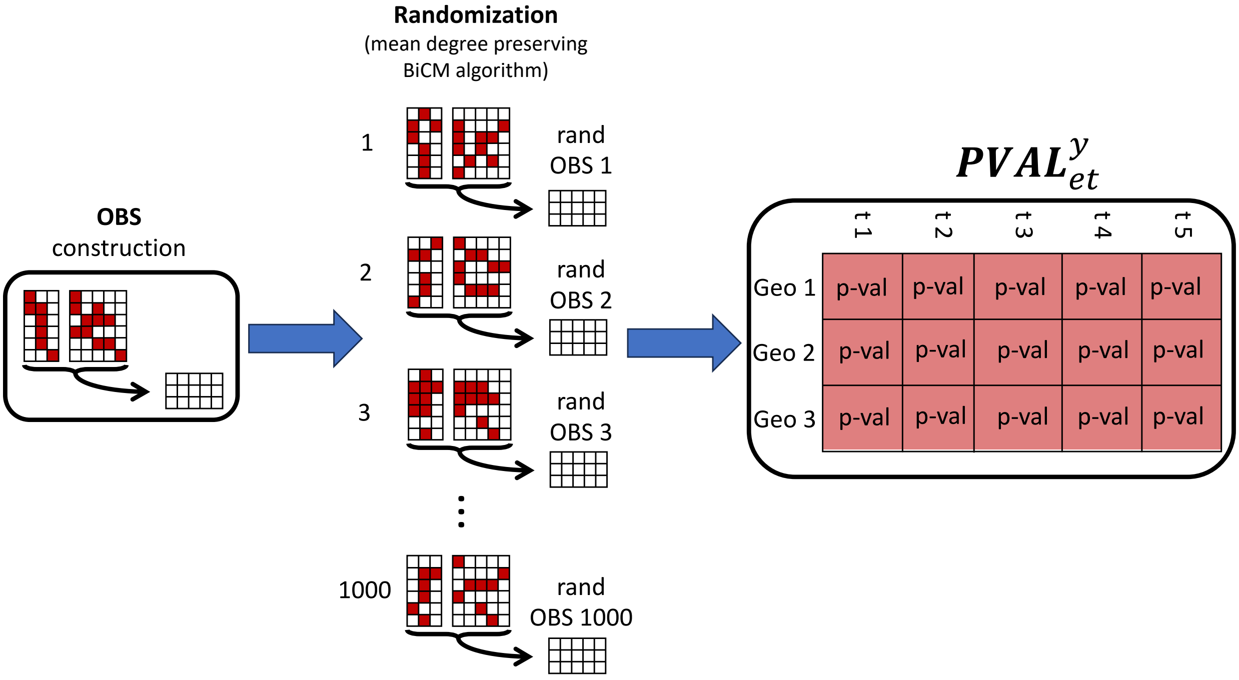

Figure 2: **Statistical validation process of $\mathbf{OBS}^y$ matrix.** After the $\mathbf{OBS}^y$ computation, we randomize 1000 times both $\mathbf{V}^y$ and $\mathbf{B}^y$ by the use of the BiCM algorithm which is used to keep the average grade of the two layers fixed. From these 1000 matrix pairs, we calculate the respective random observations and conclude by calculating the p-value of the real observations by counting how many times these are greater than the respective random observations. The final output are the $\mathbf{PVAL}^y$ matrices, which element $PVAL^y_{e,t}$ is the p-value of the $OBS^y_{e,t}$ observation.

for each 5-year time window y.

After this first process, we validate $\mathbf{OBS}^y$ matrix by comparing them against an ensemble of 1000 matrices generated using the Bipartite Configuration Model algorithm (BiCM), thus obtaining a matrix of p-values, $\mathbf{PVAL}^y$. Each element, $PVAL^y_{e,t}$, represents the p-value associated with the link between entity e and technology t for year y. This process is show in Fig. 2. The final output is the $\mathbf{PVAL}^y$ matrix, which element $PVAL^y_{e,t}$ is the p-value of the $OBS^y_{e,t}$ observation.

We conclude by binarizing $\mathbf{PVAL}^y$ using a p-value threshold of 0.05 as follows:

$$M^y_{e,t} = \begin{cases} 1 \text{ if } \mathrm{PVAL}^y_{e,t} \leq 0.05 \\ 0 \text{ if } \mathrm{PVAL}^y_{e,t} > 0.05 \end{cases}$$

and finally obtaining our $\mathbf{M}^y$ matrices.

Additional analysis of metropolitan area dual pattern

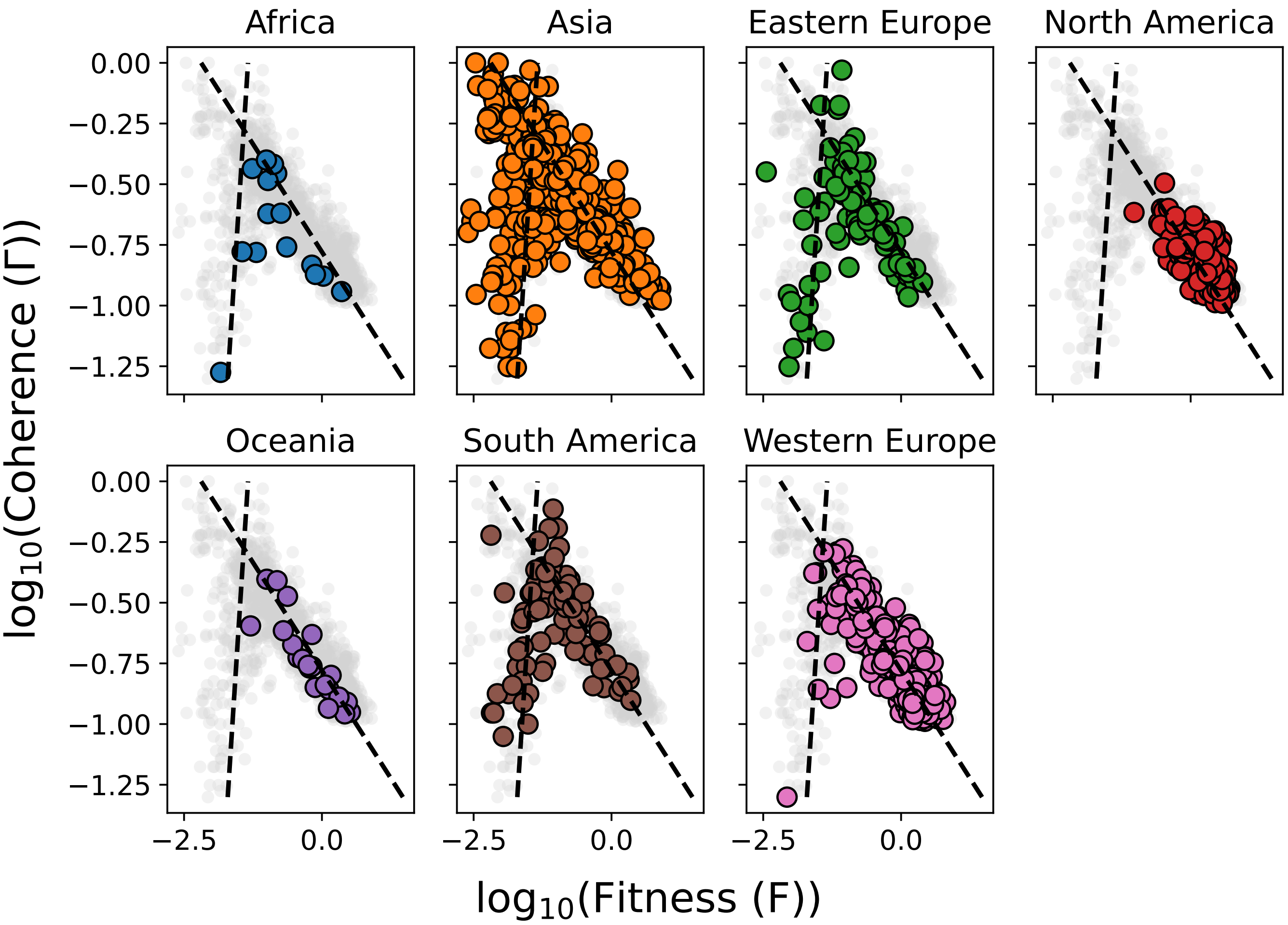

Figure 3: **Scatter plot of Fitness vs. Coherence for metropolitan areas, split into subplots by continent**. Each subplot highlights cities from its respective continent in color, with other cities shown in light grey. Western and Eastern Europe are distinguished separately. This visualization shows how continents contribute differently to the bifurcation pattern discussed in the main text.

To support the discussion of the bifurcated trend observed in the metropolitan area scatter plots (see Fig.2 in the main text), we provide two additional visualizations to clarify the geographical composition of the two branches.

- Fig. 3 shows the same Fitness vs. Coherence scatter plot for metropolitan areas, now split into separate subplots by continent. Each subplot highlights cities from its respective continent in

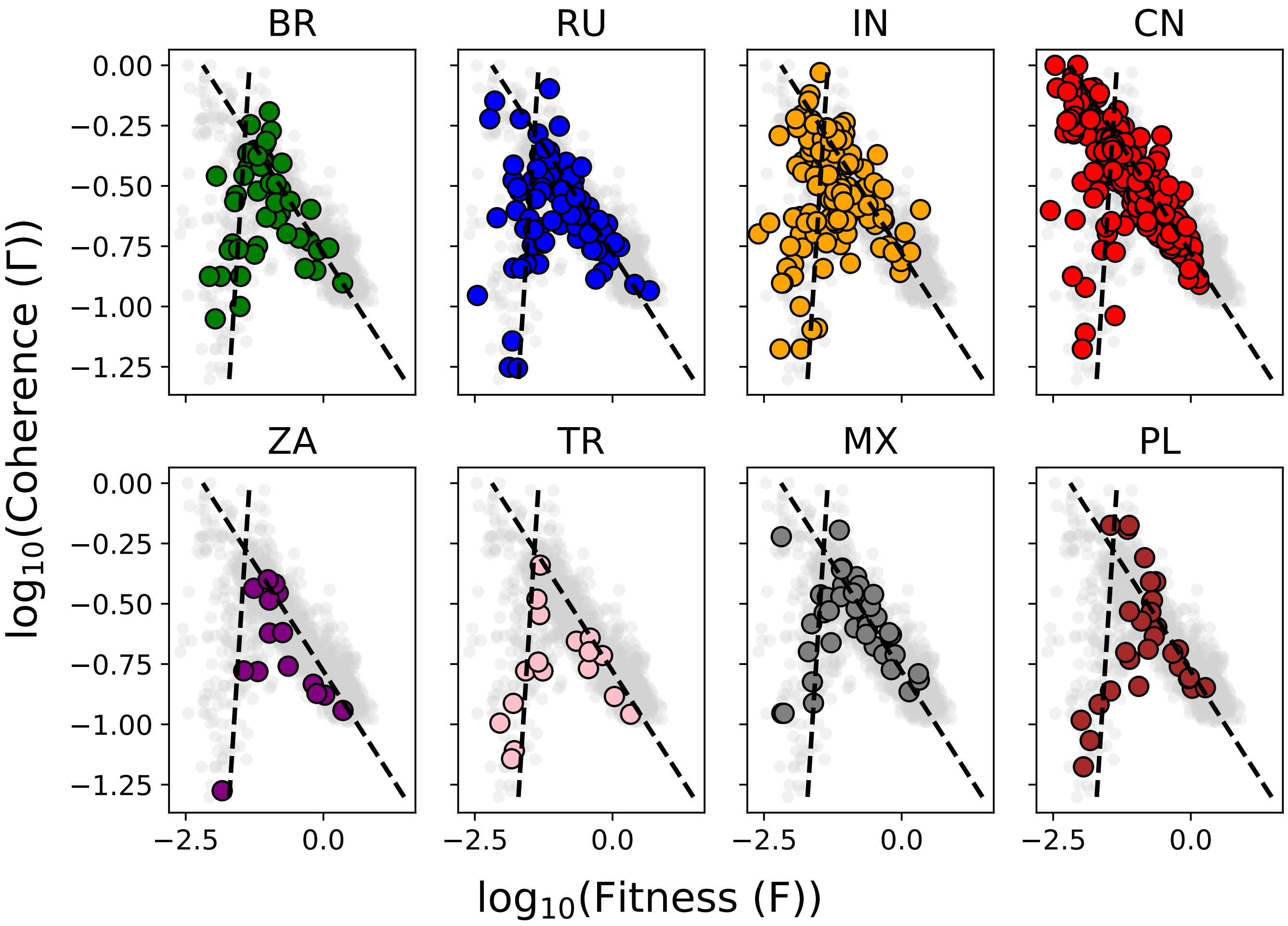

Figure 4: **Scatter plot of Fitness vs. Coherence for metropolitan areas, split into subplots by country**. Each subplot focuses on one of Brazil, Russia, India, China, South Africa, Turkey, Mexico, or Poland. Cities from the selected country are shown in color, with all other cities in light grey. This detailed view illustrates how specific countries populate the two branches, highlighting diverse innovation and growth strategies.

color, while displaying cities from other continents in light grey for context. This reveals clear clustering patterns: for example, the lower-left branch is mostly populated by South American, Asian, and Western European cities, while the tip of the lower-right branch is predominantly composed of North American and Western European cities.

- Fig. 4 shows the same Fitness vs. Coherence scatter plot for metropolitan areas, but with a focus on selected countries (Brazil, Russia, India, China, South Africa, Turkey, Mexico, and Poland). Each subplot highlights cities from a single country in color, with other cities shown in light grey. This country-level breakdown provides a clearer view of how these emerging or transitional economies contribute to shaping the bifurcation and illustrates the diversity of development paths within the general trend.

Together, these figures strengthen the argument that different clusters of metropolitan areas may follow distinct innovation and growth strategies, supporting the interpretation of dual development patterns discussed in Section 4.1 of the main text.

3 OLS regression comparison

Table 1: OLS Regression Results - Metropolitan Areas (MA) with year, MA, region, and country as fixed effects

Coefficient	With Fitness (F) & Coherence (Γ)		Without Fitness (F) & Coherence (Γ)	
	Estimate	Std. Error	Estimate	Std. Error
F	-0.0337***	0.000	–	–
Γ	0.0234***	0.002	–	–
div	0.0401***	0.001	-0.0110***	0.000
GDPpc	-0.0138***	0.000	0.0061***	0.000
Adjusted R^2: 0.708			0.464	

*** $p < 0.001$, ** $p < 0.01$, * $p < 0.05$

Table 2: OLS Regression Results - Regions with year, region, and country as fixed effects

Coefficient	With Fitness (F) & Coherence (Γ)		Without Fitness (F) & Coherence (Γ)	
	Estimate	Std. Error	Estimate	Std. Error
F	-0.0401***	0.001	–	–
Γ	-0.0013	0.005	–	–
div	0.0488***	0.001	-0.0032***	0.000
GDPpc	-0.0168***	0.000	0.0040***	0.000
year	-0.0020***	0.000	-0.0016***	0.000
Adjusted R^2: 0.498			0.336	

*** $p < 0.001$, ** $p < 0.01$, * $p < 0.05$

Table 3: OLS Regression Results - Countries with year and country as fixed effects

Coefficient	With Fitness (F) & Coherence (Γ)		Without Fitness (F) & Coherence (Γ)	
	Estimate	Std. Error	Estimate	Std. Error
F	-0.0245***	0.002	–	–
Γ	0.0121	0.029	–	–
div	0.0248***	0.002	0.0041*	0.002
GDPpc	-0.0093***	0.001	0.0004	0.001
year	-0.0019***	0.000	-0.0013***	0.000
Adjusted R^2: 0.703			0.471	

*** $p < 0.001$, ** $p < 0.01$, * $p < 0.05$

References

1. Gaétan De Rassenfosse, Jan Kozak, and Florian Seliger. Geocoding of worldwide patent data. *Scientific data*, 6(1):1–15, 2019.

2. Bela Balassa. Trade liberalisation and "revealed" comparative advantage. *The manchester school*, 33(2):99–123, 1965.

3. César A Hidalgo, Bailey Klinger, A-L Barabási, and Ricardo Hausmann. The product space conditions the development of nations. *Science*, 317(5837):482–487, 2007.

4. Andrea Zaccaria, Matthieu Cristelli, Andrea Tacchella, and Luciano Pietronero. How the taxonomy of products drives the economic development of countries. *PLoS ONE*, 9(12):e113770, 2014.

5. Emanuele Pugliese, Giulio Cimini, Aurelio Patelli, Andrea Zaccaria, Luciano Pietronero, and Andrea Gabrielli. Unfolding the innovation system for the development of countries: coevolution of science, technology and production. *Scientific Reports*, 9(1):1–12, 2019.

6. Matteo Bruno, Dario Mazzilli, Aurelio Patelli, Tiziano Squartini, and Fabio Saracco. Inferring comparative advantage via entropy maximization. *Journal of Physics: Complexity*, 4(4):045011, 2023.
